# An Evaluation of Different Digestion Methods for the Quantitation of Inorganic Elements in Human Hair Using ICP-MS

**DOI:** 10.1155/2022/5742468

**Published:** 2022-12-01

**Authors:** Yue Liu, Yang Yang, Yin-Yin Xia, Jamie V. de Seymour, De-Zhang Zhao, Yang-Mei Li, Hua Zhang, Ting-Li Han

**Affiliations:** ^1^Department of Obstetrics and Gynaecology, The Second Affiliated Hospital of Chongqing Medical University, Chongqing, China; ^2^Department of Occupational and Environmental Hygiene, School of Public Health and Management, Research Center for Medicine and Social Development, Innovation Center for Social Risk Governance in Health, Chongqing Medical University, Chongqing, China; ^3^Department of Obstetrics and Gynaecology, The First Affiliated Hospital of Chongqing Medical University, Chongqing, China; ^4^Mass Spectrometry Center of Maternal-Fetal Medicine, Chongqing Medical University, Chongqing, China; ^5^College of Health, Massey University, Auckland, New Zealand; ^6^College of Pharmacy, Chongqing Medical University, Chongqing, China; ^7^School of Public Health and Management, Research Center for Medicine and Social Development, Innovation Center for Social Risk Governance in Health, Chongqing Medical University, Chongqing, China

## Abstract

The inorganic elements have unique properties in biochemical processes in humans. An increasing number of pathologies have been associated with essential element ions, such as lead, mercury, and cadmium. Hair has become an attractive clinical specimen for studying the longitudinal exposure to elements from the external environment. Inductively coupled plasma-mass spectrometry (ICP-MS) coupled with nitric acid (HNO_3_) digestion is the most common approach for determining inorganic elements from human hair. This study aims to optimize the digestion method for the absolute quantitation of 52 elements using ICP-MS, for a large cohort study in human hair. Five different HNO_3_ (65%) digestion methods were investigated and evaluated for their internal standard solution stability, reproducibility, element coverage, and standard solution recovery efficiency, namely, room temperature for 24 h (RT), 90°C for 4 h (T90), ultrasonic-assisted digestion (UltraS), programmed digestion of microwave digestion (MicroD), and ordinary microwave oven digestion (O-MicroD). Our results demonstrated that O-MicroD, MicroD, and RT were the best performing digestion methods for coefficient of variation (CV) scores, coverage, and recovery efficiency, respectively. In particular, the O-MicroD method detected multiple elements in a small quantity of hair (3 mg), with minimum nitric acid usage (200 *μ*l) and a short digestion time (30 min). The O-MicroD method had excellent reproducibility, as demonstrated by a continuous thousand injections of hair samples with three internal standards (CV: ^103^Rh = 3.59%, ^115^In = 3.61%, and ^209^Bi = 6.31%). Future studies of the elemental content of hair should carefully select their digestion method to meet the primary purpose of their study.

## 1. Introduction

Inorganic elements have unique properties that cannot be performed by organic compounds. Some elements are indispensable for humans and essential in biochemical processes. For example, iron (Fe), copper (Cu), and manganese (Mn) are all positioned at the metal-catalytic site of enzymes involved in redox reactions [1]. Calcium, magnesium, and sodium maintain the electrochemical gradient of the cell membranes [1]. There are many metal ions that are important for cellular metabolism and mitochondrial function, such as K^+^, Mg^2+^, and Zn^2+^ [2]. Nevertheless, growing evidence suggests that excessive exposure to some elements could cause adverse health outcomes. The role of cadmium (Cd), mercury (Hg), and copper (Cu) in type 2 diabetes, renal dysfunction, cardiovascular diseases, osteoporosis, and cancers, has been reported in experimental and epidemiological studies [3–5]. Excessive aluminium (Al), lead (Pb), and arsenic (As) exposures have also been associated with oxidative stress, intestinal diseases, dyslipidemia, and metabolic diseases [3, 6, 7]. Exposure to metals can be mediated through food, dermal contact, air pollution, and drinking water [7]. Elements absorbed by the pulmonary tract and the digestive system can be distributed via the bloodstream to a range of different organs and also to the hair [8]. Furthermore, some elements might be deposited on the hair through external contamination. Hence, measurements of the composition of the hair can reflect both endogenous and exogenous exposures to various inorganic elements.

Hair is a proteinaceous fibre predominantly consisting of keratin proteins [9]. Most inorganic elements have a high affinity for the sulfhydryl group of amino acids in keratin. Therefore, inorganic elements are easily incorporated and retained in human hair [9]. The accumulation of inorganic elements in hair reflects long-term exposure. Since metal concentrations are reduced in urine and blood after days and weeks, respectively, human hair appears to be a more robust specimen for estimating past and ongoing exposure to inorganic elements. Hair also has less background matrix than urine and blood, and the inorganic elements are easier to detect analytically because they are usually present at higher levels in the hair. Moreover, hair samples are collected noninvasively and can be transported and stored without refrigeration or prior processing. Hence, hair has become an attractive clinical specimen for studying the longitudinal exposure of inorganic elements from the external environment.

Numerous instruments have been employed for element detection and quantitation, including inductively coupled plasma-mass spectrometry (ICP-MS), inductively coupled plasma-optical emission spectrometry (ICP-OES), instrumental neutron activation analysis (INAA), electrothermal-atomic absorption spectrometry (ET-AAS), and flame atomic absorption spectrometry (FAAS) [10–14]. For qualitative and quantitative measurement of hair elements, ICP-MS is our selected platform because it has the most superior sensitivity, resolving power, and improved limits of detection (LODs) to measure elements [8]. A variety of hair digestion techniques for element detection via ICP-MS analysis have been published, including using a microwave digestion instrument, leaving the sample at room temperature for 24 h, using a heat block, and using an ultrasonic water bath [15–19]. These approaches are generally coupled with efficient acid decomposition procedures such as digestion in concentrated nitric acid (65–68%), and only a few studies use other solutions [8, 20, 21]. However, these methods often use a large amount of hair biomass (30–150 mg), excessive acid solution (3–10 ml per sample), and extensive digestion time (1 hr–2 days) [15–19]. These factors are not conducive to the efficient analysis of a large cohort of samples, and not all participants are willing to donate a large volume of hair. It is clear that the methodology for analysing inorganic elements in hair needs to be improved in order to effectively and efficiently analyse hair element concentrations in large cohorts.

This study aims to establish a method for analysing the inorganic elements in human hair samples from a large cohort, using low hair mass. We analysed five different sample preparation methods for their reproducibility, element coverage, and extraction efficiency. The results from our findings have informed the methodology for a large cohort study of hair from pregnant women to investigate the link between exposure to inorganic elements and pregnancy/fetal outcomes.

## 2. Experimental

### 2.1. Hair Sample Collection

Hair samples were collected from pregnant women in the Complex Lipids in Mothers and Babies (CLIMB) cohort (Chinese Clinical Trial Register number: ChiCTR-IOR-16007700) [22]. The 3–6 hair strands were taken from the occipital area, 0.5 cm away from the scalp. The hair samples were cut into pieces and stored in a self-sealing bag at −20°C. The collection and segmentation of hair were done with scissors made of polytetrafluoroethylene (PTFE) to avoid elemental contamination. A thousand hair samples collected from CLIMB were used to test the reproducibility of the chosen method. These hair samples were collected in accordance with the method published by Delplancke et al. [23] and in full agreement with the principles of the International Conference on Harmonisation Good Clinical Practice E6 (ICH-GCP) and the Declaration of Helsinki. The study was approved by the Ethics Committee of Chongqing Medical University (No.2014034), and written informed consent was obtained from all participants.

### 2.2. Reagents and Calibration Standard Solutions

Analytical and internal standards were purchased from Agilent Technologies. ICP-MS-grade nitric acid (65%) and acetone were obtained from ANPEL Laboratory Technologies (Shanghai, China). Ultrapure deionized water (18 mΩ) was obtained from a water purification system (Aoside, China).

A working standard solution was prepared by diluting 10 *μ*g/mL of mixed-element standard solutions (Multielement Calibration Standard 2A: Ag, Al, As, Ba, Be, Ca, Cd, Co, Cr, Cs, Cu, Fe, Ga, K, Li, Mg, Mn, Na, Ni, Pb, Rb, Se, Sr, Tl, U, V, and Zn; Multielement Calibration Standard 4: B, Ge, Mo, Nb, P, Re, S, Si, Ta, Ti, W, and Zr; Multielement Calibration Standard 1: Ce, Dy, Er, Eu, Gd, Ho, La, Lu, Nd, Pr, Sc, Sm, Tb, Th, Tm, Y, and Yb) or a single-element standard solution (Multielement Calibration Standard 2A-HG: Hg). Two sets of seven standard solutions for calibration curves were prepared. The first set contained all the elements (except Hg) diluted in 5% HNO_3_ in 15 ml centrifuge tubes (concentration gradient: 0.0 *μ*g/L, 0.1 *μ*g/L, 0.5 *μ*g/L, 2.5 *μ*g/L, 25 *μ*g/L, 250 *μ*g/L, and 2500 *μ*g/L). The second set contained the low concentration Hg (<5 *μ*g/L) diluted in 5% HNO_3_ in 15 ml centrifuge tubes (concentration gradient: 0.0 *μ*g/L, 0.02 *μ*g/L, 0.1 *μ*g/L, 0.5 *μ*g/L, and 2.5 *μ*g/L). An internal standard (IS) solution was also prepared by diluting 100 *μ*g/mL ICP-MS Internal Standard Mix in 5% HNO_3_ (Bi, Ge, In, Li, Lu, Rh, Sc, and Tb) (ICP-MS Internal Standard Mix: 5% HNO_3_ = 1 : 200).

### 2.3. Hair Digestion and Element Extraction Methods

Five different extraction methods were chosen based on previously reported studies analysing human hair acid decomposition. The extraction methods were as follows: (1) HNO_3_ digestion at room temperature for 24 h (RT), (2) HNO_3_ digestion at 90°C for 4 h in a constant temperature drying oven (T90) (Thermo Scientific), (3) ultrasonic-assisted acid digestion with HNO_3_ (UltraS) (SCIENTZ, SB-5200DT), (4) programmed digestion using microwave digestion with HNO_3_ (MicroD, Table S1) (PreeKem, WX-6000), and (5) ordinary microwave oven digestion with HNO_3_ (O-MicroD, 100% power, 30 min) (Galanz, G70D20CN1P-D2(S0)).

### 2.4. Sample Preparation

The hair strands were washed two times with acetone and then once with deionized water, prior to drying in an oven at 37°C. Hair segments were mixed together in 2 mL eppendorf tubes with PTFE beads to break the hairs into powder using tissue lysis П (QIAGEN), to ensure homogeneity of the hairs for all digest methods under investigation. Four replicates of 3 mg ± 0.5 mg of mixed hair were weighed into 15 mL PTFE digestion tubes. Next, 200 *μ*L of concentrated nitric acid was added, and the tubes were capped to carry out the digestion reaction. Importantly, the MicroD digestion method required adding 3 mg ± 0.5 mg hair sample and 5 ml concentrated nitric acid to the matching digestion tank and then concentrated the acid into 200 *μ*l before transfer. After the samples had dissolved, they were transferred to 15 mL centrifuge tubes and made up to a volume of 3 mL with deionized water. A 100 *μ*l aliquot of each of the three mixed standard solutions was transferred into the PTFE tube, and then 200 *μ*l of the concentrated nitric acid solution was added. After digestion, using the five different methods detailed above, the digested solution was transferred to 15 ml centrifuge tubes and made up to a volume of 10 mL with deionized water.

### 2.5. Inductively Coupled Plasma-Mass Spectrometry (ICP-MS) Analysis

The samples were handled using a SPS 4 autosampler (Agilent Technologies). The ICP-MS/MS instrument was an Agilent 8900 controlled with MassHunter 4.6 Workstation Software 8900 ICP-QQQ Top (C.01.06). The 8900 ICP-MS/MS uses a tandem mass spectrometer layout, with two quadrupole mass spectrometers enabling it to operate in the MS/MS mode. ICP-QQQ offers an additional quadrupole mass filter in front of the collision reaction cell to resolve spectral interferences. The 8900 has higher sensitivity and a specialized flow path with argon gas to provide lower backgrounds (sensitivity: ^59^Co ≥ 22 × 106 cps/ppm (<10% RSD, relative standard deviation) and background (on mass) mass 78 ≤ 400 cps). The operating conditions (lenses, torch position, detector voltage, and gas flows) were optimized daily (the count of mass 7, 89, and 205 needs to reach more than 3000, 10000, and 6000, respectively; oxide <2%; doubly charged <3%; peak width -10%: 0.65–0.80), using a 1 *μ*g/L tuning solution (Ce, Co, Li, Mg, Tl, and Y; Agilent Technologies). The main ICP-MS detection parameters were as follows: radio-frequency (RF) power: 1550.0 w, RF matching: 1.80 V, sampling depth: 10.0 mm, nebulizer gas flow: 1.05 L/min, peristaltic pump speed: 0.1 rps, atomization chamber temperature: 2°C, extract 2: −230.0 V, omega bias: −105.0 V, omega lens: 8.8 V, collision cell entrance: −50.0 V, collision cell exit: −60.0 V, Q1 entrance: −50.0 V, Q1 exit: 1.0 V, Q1 bias: −1.0 V, flow rate of argon cell for collision cell: 5.5 mL/min, OctP RF: 150.0 V, and OctP bias: −18.0 V. An internal standard solution was online to join by peristaltic pump.

### 2.6. Elemental Quantitation, Normalization, and Statistical Analysis

Inorganic element identification and absolute quantitation were performed using MassHunter 4.6 Workstation Software 8900 ICP-QQQ Data Analysis (C.01.06). The calibration standard's regression equation and correlation coefficient (*r*) for individual elements were determined (Table S2). Only elements not in the calibration standard solution were selected as ISs (^103^Rh, ^115^In, ^209^Bi). The measuring elements were normalized by the most appropriate IS according to the following mass range: ^103^Rh for mass 23–107, ^115^In for mass 111–169, and ^209^Bi for mass 172–238. The blank deduction was performed by subtracting the blank from the sample results. Calibration curves were then set up by external calibration, linear fit, and blank offset. The *y* value was obtained by dividing the count of each point of the correction curve by the count of the unit concentration of the internal standard of the same level as follows:

(1) $$\begin{array}{c} y=\frac{y_{\sigma }}{\left(y_{i}/x_{i}\right)}\text{,} \end{array}$$

when *x*_*i*_ = 0, *y* = *y*_*σ*_; *x*_*i*_ is the IS element concentration, *y*_*i*_ is the IS element count, and *y*_*σ*_ is the sample data count.

The correlation coefficient of the linear regression correction curve was calculated using the following formula:

(2) $$\begin{array}{c} r=\frac{\sum_{i}\left\{\left(x_{i}-\bar{x}\right)\left(y_{i}-\bar{y}\right)\right\}}{{\left\{\left[{\sum_{i}\left(x_{i}-\bar{x}\right)}^{2}\right]\left[{\sum_{i}\left(y_{i}-\bar{y}\right)}^{2}\right]\right\}}^{1/2}}\text{,} \end{array}$$

where $\bar{x}$ is the average of *x*_*i*_, $\bar{y}$ is the average of *y*_*i*_, *x*_*i*_ is the determination value of *x*, and *y*_*i*_ is the determination value of *y*.

The relative error (%RE) was calculated according to the following formula:

(3) $$\begin{array}{c} \%RE=\frac{x_{i}^{\prime }-x_{i}}{x_{i}}\times 100\text{,} \end{array}$$

where *x*_*i*_ is the actual value of the calibration standard and *x*_*i*_′ is the concentration of the collected corrected standard.

The relative standard error (%RSE) represented the fitting index of the correction curve, a value calculated using the following formula:

(4) $$\begin{array}{c} \%RSE=100\times \sqrt{\frac{{\sum_{i-1}^{n}\left[\left(x_{i}^{\prime }-x_{i}\right)/x_{i}\right]}^{2}}{\left(n-p\right)}}\text{,} \end{array}$$

where *x*_*i*_ is the actual value of the calibration curve level *i*, *x*_*i*_′ is the collection concentrations of the calibration curve level *i*, *p* is the number of items for the calibration curve formula, and *n* is the number of points of the available calibration curve.

Coefficient of variance (CV) and principal component analysis (PCA) were used to check the repeatability of digestion methods. Boxplots, two-dimensional projections of the 3D PCA, bar graphs, dot-line graphs, and scatter plots were rendered using the ggplot2 R package. Element coverage was compared across the five digestion protocols and displayed using an UpSet plot, constructed using the UpSet *R* package. The cumulative coefficient of variation was illustrated by a horizontal bar chart constructed in Microsoft Excel version 2019. The recovery efficiency of elements in the standard solution was compared and displayed using a Microsoft Excel bar chart.

## 3. Results and Discussion

This study was the first to assess five different HNO_3_ hair digestion methods in preparation for inorganic element analysis using ICP-MS. A total of 52 elements were quantitated in this study. The identified elements were subdivided into eight major metal classes such as alkali metals, alkaline Earth metals, transition metals, and others (Table S3). Their detailed concentrations are displayed in Table S4. Metals detected in each of the classes were evaluated in detail to assess the performance of the five different digestion methods. The performance of each method was evaluated by testing internal standard solution stability, digestion reproducibility, metal coverage, and standard solution recovery efficiency.

### 3.1. Internal Standard Solution Stability of the Five Different Digestion Methods

The internal standard solution stability of the five selected methods was evaluated by comparing levels of the IS elements (Bi, In, and Rh) in the hair samples following the different digestion methods (Figure S1). The IS stability of all digestion methods was between 80% and 120%, which meets the requirements of the instrument measurement. None of the IS elements drifted as an outlier in the O-MicroD, RT, and UltraS methods, while there were abnormally high outliers in the T90 and MicroD methods. The RT method demonstrated the best IS stability in this study.

### 3.2. Digestion Recovery Efficiency of the Standard Solution of Five Different Digestion Methods

**Figure 1:**
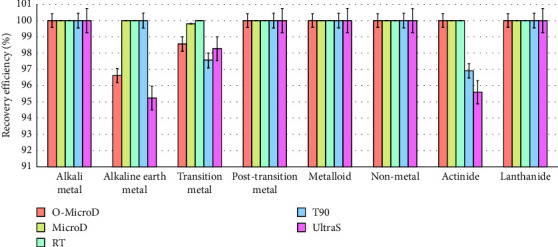
The bar chart represents the recovery efficiency (%) of eight different element classes across five different digestion methods for the standard solution.

The first criterion which was used to evaluate the accuracy of the five different digestion methods was recovery efficiency of the standard solution. This is determined according to the recovery levels of different elements in a digested standard solution of a known concentration, as shown in Figure 1. The RT and MicroD methods displayed the 99%–100% recovery efficiency for all eight different element classes from the standard solution; O-MicroD only produced the high recovery yield for alkali metals, post-transition metals, metalloids, nonmetals, lanthanide, and actinide; T90 only performed the high recovery for alkali metals, alkaline Earth metals, post-transition metals, metalloids, nonmetals, and lanthanide; UltraS was superior for alkali metals, post-transition metals, metalloids, nonmetals, and lanthanide. We hypothesized that the RT method displayed the best recovery efficiency because it only involves two sample preparation steps (dilution and incubation) and an extensive 24 h digestion period. In addition, the MicroD method also exhibited high recovery efficiency and this is likely because electromagnetic radiation can penetrate through the hair matrix, promoting molecular rotations and efficient heating via friction [24]. Microwave digestion has been found to be one of the most convenient techniques to prepare samples for elemental determination [25–27].

### 3.3. Reproducibility of the Five Different Digestion Methods

**Figure 2:**
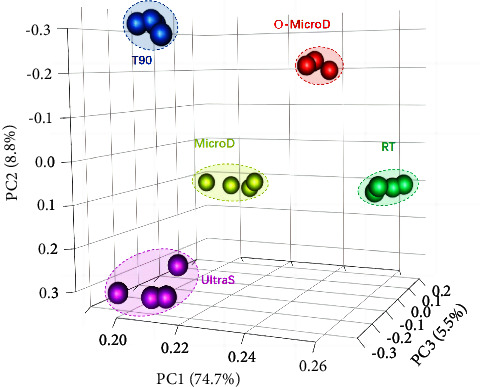
Principal component analysis (PCA) of the human hair elements, digested using five different methods. Each color represents a digestion method. An ellipse with dotted lines indicates the 95% confidence interval calculated using Hotelling's T2 statistics.

**Figure 3:**
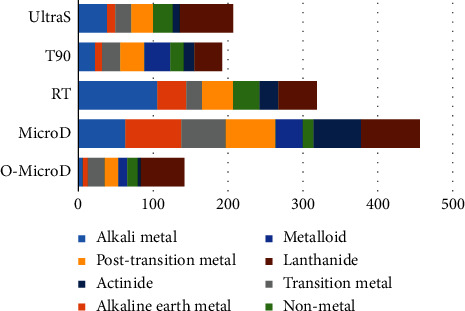
The horizontal bar chart represents the cumulative coefficient of variations (%) of eight different metal classes across five different digestion methods for human hair. The *x*-axis is the total CV for all major metal classes.

**Figure 4:**
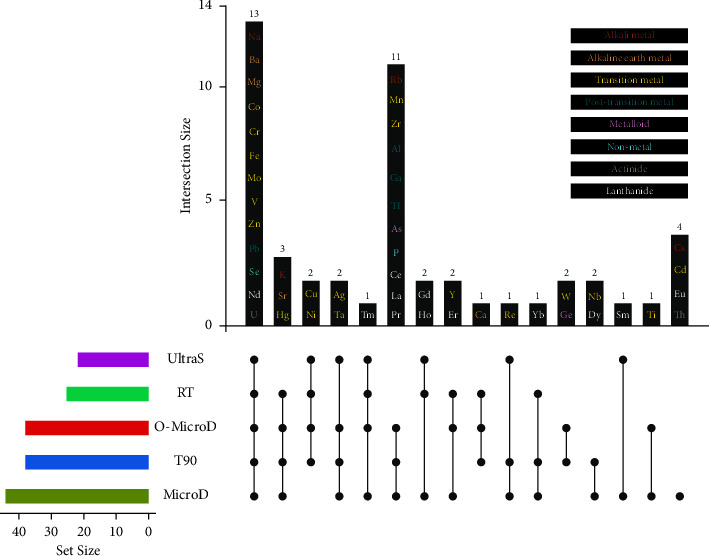
Upset plot of the identified metal coverage compared across five different digestion methods for human hair.

The O-MicroD method demonstrated the most promising digestion reproducibility for the trace amount of human hair analysed. The PCA analysis showed that the technical replicates of the O-MicroD hair elements (red dots) were clustered the closest to each other along PC1 (74.7%), PC2 (8.8%), and PC3 (5.5%) (Figure 2). Consistent with the results of the PCA, the O-MicroD digestion method exhibited the most reproducible CV (CV: 23.49%) for the overall metal classes identified (Figure 3, Table S3 and Figure S2). The MicroD digestion method displayed the least reproducible CV for hair (CV: 64.37%). The reproducibility of lanthanide was poor across all five digestion methods, with a minimum CV of 36.87% (T90) (Figure 3, Table S3). In addition, we found that the hair samples that were digested using the MicroD method exhibited the highest number of elements with poor reproducibility (Figure 4). The possible explanation for the O-MicroD digestion method possessing superior reproducibility for most metal elements in a trace amount of human hair could be that the microwave has heating stability and less volume is lost during solution transfer. In contrast, the MicroD method showed the poorest reproducibility due to additional preparation steps, that is, the microwave-digested hair mixture was further heated and evaporated from 5 ml to 0.2 ml to eliminate the high HNO_3_ concentration prior to ICP-MS analysis. This significant fluctuation in volume may greatly increase the variability of element concentrations among individual samples.

### 3.4. Element Coverage of the Five Different Digestion Methods

**Figure 5:**
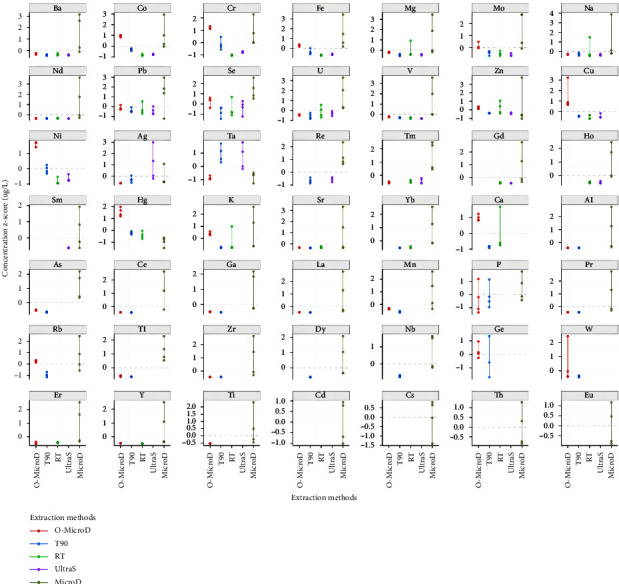
The dot-line graphs show the element concentrations of each sample across five different digestion methods for human hair. Each dot represents a sample concentration. The vertical lines are the standard deviation of each digestion method. The metal concentrations were standardized to *Z*-scores.

Another criterion that we used to determine the optimal digestion method was the element coverage. The order of digestion methods ranking from the highest to lowest number of identified elements in human hair were as follows: MicroD (44 elements), O-MicroD (38 elements), T90 (38 elements), RT (25 elements), and UltraS (22 elements), as displayed in Table S3. Noticeably, our microwave digestion approaches have quantified more elements simultaneously in the hair matrix than in other studies; Hao et al. identified 15 hair elements using HNO_3_/H_2_O_2_ at 100°C until dryness using an electric heating plate [28]; Luo et al. quantified 16 hair elements using HNO_3_ and H_2_O_2_ digestion at 90°C for 3 h prior to ICP-MS analysis [16]; Tinkov et al. reported 17 trace elements from children's hair using microwave digestion (170–180°C) with HNO_3_ [29]. We propose that this has been achieved by the following two strategies: (1) we have established calibration curves for 56 elements in a single standard dilution series by combining three sets of multielement calibration solutions; (2) the Agilent 8900 ICP-MS used in our study exhibits high sensitivity and strong antispectral interference capability through higher mass spectrum scanning frequency (3.0 MHz) and an additional quadrupole mass filter. Furthermore, the Upset plot and dot-line graphs illustrate that there are 13 elements that can be effectively digested and identified by using any of the five methods (Figures 4 and 5: Na, Ba, Mg, Co, Cr, Fe, Mo, V, Zn, Pb, Se, Nd, and U), 11 elements that can be effectively identified by using three of the tested methods (Figures 4 and 5: O-MicroD, MicroD, T90 : Rb, Mn, Zr, Al, Ga, Tl, As, P, Ce, La, and Pr), and four elements which can only can be identified by using the MicroD method (Figures 4 and 5: Cs, Cd, Eu, and Th). Overall, the O-MicroD and MicroD methods showed the most comprehensive coverage of identified elements in this study.

**Figure 6:**
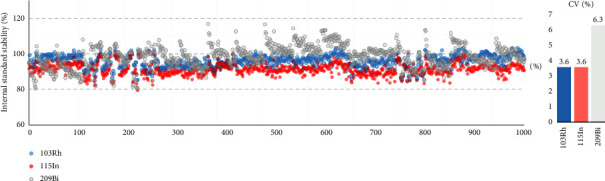
The scatter plot and coefficient of variation (CV) for the internal standard stability from the ICP-MS hair analysis of a thousand maternal hair samples using O-MicroD digestion.

Since the O-MicroD method could reproducibly quantitate a large number of elements using only 3 mg of hair biomass with 200 *μ*l of nitric acid for 30 min digestion time, we further tested the reproducibility of O-MicroD by coinjecting three IS with a thousand hair samples. We found that the CV of ^103^Rh, ^115^In, and ^209^Bi were 3.59%, 3.61%, and 6.31%, respectively, without batch correction (Figure 6). The excellent reproducibility of O-MicroD, together with minimizing sample usage and preparation time, makes this approach an ideal choice for a large cohort studies.

In summary, our findings suggest that out of the five digestion methods tested, the O-MicroD method had the best reproducibility and digestion time (30 mins) for elemental analysis of human hair, the MicroD method had the best element coverage, and the RT method performed best in element digestion recovery efficiency.

## 4. Conclusion

In conclusion, this is the first study conducted to investigate the optimal digestion method for analysing the 52 inorganic elements in human hair using ICP-MS analysis. Based on the criteria of reproducibility, detected element coverage, and the digestion recovery yield, the O-MicroD, MicroD, and RT methods were the superior digestion methods for CV, coverage, and recovery efficiency, respectively. However, we also discovered that different digestion methods favor the digestion of different element classes. Future studies should carefully consider the digestion method they select, choosing the method that would be best suited to the primary purpose of their study.

## Data Availability

The data used to support the results of this study are included within the article. Any further information is available from corresponding authors upon request.

## Abbreviations

CLIMB: Complex Lipids in Mothers and Babies
CV: Coefficient of variation
ET-AAS: Electrothermal-atomic absorption spectrometry
FAAS: Flame atomic absorption spectrometry
ICP-MS: Inductively coupled plasma-mass spectrometry
ICP-OES: Inductively coupled plasma-optical emission spectrometry
INAA: Instrumental neutron activation analysis
LOD: Limits of detection
MicroD: Programmed digestion of microwave digestion
O-MicroD: Ordinary microwave oven digestion
PCA: Principal component analysis
PTFE: Polytetrafluoroethylene
RE: Relative error
RSE: Relative standard error
RT: Room temperature for 24 h
T90: 90°C for 4 h
UltraS: Ultrasonic assisted digestion.

## Supplementary Materials

Supplementary Materials Figure S1: the extraction efficiency of internal standard elements (Bi, In, and Rh) in five different digestion methods for human hair. Figure S2: the bar graphs show the coefficient of variation (%) of each metal across five different digestion methods for human hair. Table S1: microwave digestion procedure parameters. Table S2: linear relationship and detection limit of each element in the standard solution. Table S3: number of identified elements, concentrations (*μ*g/L), and CVs (%) of each element classes in human hair, compared across five different digestion methods. Table S4: the quantitative concentrations (*μ*g/L) of identified metals in human hairs across the five different digestion methods.
