# Supplementary material for: An Evaluation of Different Digestion Methods for the Quantitation of Inorganic Elements in Human Hair Using ICP-MS
Source: J Anal Methods Chem. 2022 Dec 1;2022:5742468. doi: 10.1155/2022/5742468 (PMC9731750; doi:10.1155/2022/5742468)
Supplement: Supplementary Materials — Figure S1: the extraction efficiency of internal standard elements (Bi, In, and Rh) in five different digestion methods for human hair. Figure S2: the bar graphs show the coefficient of variation (%) of each metal across five different digestion methods for human hair. Table S1: microwave digestion procedure parameters. Table S2: linear relationship and detection limit of each element in the standard solution. Table S3: number of identified elements, concentrations (μg/L), and CVs (%) of each element classes in human hair, compared across five different digestion methods. Table S4: the quantitative concentrations (μg/L) of identified metals in human hairs across the five different digestion methods. [file 5742468.f1.docx]

| **Table S1. Microwave digestion procedure parameters.** | | | |
| --- | --- | --- | --- |
| **Steps** | **Temperature/℃** | **Maintain Time/min** | **Pressure/atm** |
| 1 | 100 | 6 | 15 |
| 2 | 150 | 7 | 25 |
| 3 | 170 | 8 | 35 |
| 4 | 190 | 9 | 40 |

| **Table S2. Linear relationship and detection limit of each element in the standard solution.** | | | | | |
| --- | --- | --- | --- | --- | --- |
| Elements | Classes | IS | Equation of linear regression | r | LOD (μg/L) |
| Cs | Alkali metal | ^115^In | y = 2.024E-2 x + 2.305E-4 | 1.0000 | 0.0129 |
| K | Alkali metal | ^103^Rh | y = 8.531E-5 x + 5.166E-3 | 1.0000 | 5.5670 |
| Na | Alkali metal | ^103^Rh | y = 2.143E-4 x + 2.288E-3 | 0.9998 | 3.1460 |
| Rb | Alkali metal | ^103^Rh | y = 1.141E-3 x + 1.361E-5 | 1.0000 | 0.0247 |
| Ba | Alkaline earth metal | ^115^In | y = 4.416E-3 x + 2.567E-4 | 1.0000 | 0.0250 |
| Ca | Alkaline earth metal | ^103^Rh | y = 4.828E-5 x + 2.061E-4 | 0.9999 | 1.5210 |
| Mg | Alkaline earth metal | ^103^Rh | y = 9.878E-5 x + 4.909E-5 | 1.0000 | 0.4305 |
| Sr | Alkaline earth metal | ^103^Rh | y = 1.426E-3 x + 1.718E-4 | 1.0000 | 0.0627 |
| Ag | Transition metal | ^103^Rh | y = 1.221E-2 x + 2.033E-4 | 1.0000 | 0.0095 |
| Cd | Transition metal | ^115^In | y = 6.311E-3 x + 6.861E-5 | 1.0000 | 0.0127 |
| Co | Transition metal | ^103^Rh | y = 7.581E-3 x + 1.181E-5 | 1.0000 | 0.0006 |
| Cr | Transition metal | ^103^Rh | y = 3.910E-3 x + 1.688E-4 | 1.0000 | 0.0401 |
| Cu | Transition metal | ^103^Rh | y = 6.514E-3 x + 3.303E-3 | 1.0000 | 0.2042 |
| Fe | Transition metal | ^103^Rh | y = 2.948E-3 x + 1.688E-3 | 0.9999 | 0.0625 |
| Hg | Transition metal | ^209^Bi | y = 1.872E-3 x + 2.787E-5 | 0.9921 | 0.0250 |
| Mn | Transition metal | ^103^Rh | y = 1.301E-3 x + 2.177E-5 | 1.0000 | 0.0377 |
| Mo | Transition metal | ^209^Bi | y = 3.205E-3 x + 1.636E-5 | 1.0000 | 0.0001 |
| Ni | Transition metal | ^103^Rh | y = 2.294E-3 x + 2.162E-4 | 1.0000 | 0.0328 |
| Re | Transition metal | ^209^Bi | y = 1.532E-2 x + 1.740E-5 | 1.0000 | 0.0017 |
| Sc | Transition metal | ^103^Rh | y = 6.084E-4 x + 2.123E-2 | 1.0000 | 2.2740 |
| Ta | Transition metal | ^209^Bi | y = 6.116E-2 x + 1.479E-4 | 1.0000 | 0.0008 |
| Ti | Transition metal | ^103^Rh | y = 5.476E-5 x + 2.727E-6 | 1.0000 | 0.2588 |
| V | Transition metal | ^103^Rh | y = 2.729E-3 x + 2.722E-6 | 1.0000 | 0.0001 |
| W | Transition metal | ^209^Bi | y = 1.583E-2 x + 3.014E-4 | 1.0000 | 0.0049 |
| Y | Transition metal | ^103^Rh | y = 4.168E-3 x | 1.0000 | 0.0001 |
| Zn | Transition metal | ^103^Rh | y = 7.486E-4 x + 5.158E-4 | 1.0000 | 0.0908 |
| Zr | Transition metal | ^103^Rh | y = 4.771E-3 x + 7.099E-5 | 1.0000 | 0.0197 |
| Nb | Transition metal | ^103^Rh | y = 1.226E-2 x + 1.364E-5 | 1.0000 | 0.0023 |
| Al | Basic-metal | ^103^Rh | y = 1.823E-5 x + 5.634E-5 | 0.9998 | 2.1140 |
| Ga | Basic-metal | ^103^Rh | y = 7.584E-4 x | 1.0000 | 0.0001 |
| Pb | Basic-metal | ^209^Bi | y = 8.302E-3 x + 1.842E-3 | 1.0000 | 0.0113 |
| Tl | Basic-metal | ^209^Bi | y = 3.106E-2 x + 1.637E-5 | 1.0000 | 0.0007 |
| As | Metalloid | ^103^Rh | y = 4.922E-4 x + 7.087E-6 | 1.0000 | 0.0105 |
| Ge | Metalloid | ^103^Rh | y = 6.972E-4 x + 2.086E-2 | 1.0000 | 0.6008 |
| P | Non-metal | ^103^Rh | y = 1.496E-6 x + 2.722E-5 | 0.9998 | 33.9500 |
| Se | Non-metal | ^103^Rh | y = 4.237E-5 x + 1.091E-6 | 1.0000 | 0.0338 |
| Ce | Lanthanide | ^115^In | y = 9.616E-2 x + 4.603E-4 | 1.0000 | 0.0031 |
| Er | Lanthanide | ^115^In | y = 5.479E-2 x | 1.0000 | 0.0001 |
| La | Lanthanide | ^115^In | y = 7.149E-2 x + 2.734E-5 | 1.0000 | 0.0020 |
| Nd | Lanthanide | ^115^In | y = 1.690E-2 x | 1.0000 | 0.0001 |
| Pr | Lanthanide | ^115^In | y = 8.625E-2 x + 1.367E-5 | 1.0000 | 0.0008 |
| Tm | Lanthanide | ^115^In | y = 1.805E-1 x | 1.0000 | 0.0001 |
| Dy | Lanthanide | ^115^In | y = 3.680E-2 x | 1.0000 | 0.0001 |
| Eu | Lanthanide | ^115^In | y = 5.654E-2 x + 1.361E-5 | 1.0000 | 0.0013 |
| Gd | Lanthanide | ^115^In | y = 3.081E-2 x | 1.0000 | 0.0001 |
| Tb | Lanthanide | ^116^In | y = 1.598E-1 x + 5.787E0 | 1.0000 | 1.6590 |
| Ho | Lanthanide | ^115^In | y = 1.570E-1 x | 1.0000 | 0.0001 |
| Sm | Lanthanide | ^115^In | y = 1.389E-2 x | 1.0000 | 0.0001 |
| Yb | Lanthanide | ^209^Bi | y = 8.579E-3 x | 1.0000 | 0.0001 |
| Lu | Lanthanide | ^210^Bi | y = 7.852E-2 x + 2.888E0 | 1.0000 | 1.9850 |
| Th | Actinide | ^209^Bi | y = 3.891E-2 x + 2.336E-5 | 1.0000 | 0.0017 |
| U | Actinide | ^209^Bi | y = 4.478E-2 x + 1.722E-5 | 1.0000 | 0.0015 |
| IS: internal standard, LOD: limit of detection. | | | | | |

| **Table S3. Number of identified elements, concentrations (μg/L) and CVs (%) of each element classes in human hair, compared across five different digestion methods.** | | | | | | | | | |
| --- | --- | --- | --- | --- | --- | --- | --- | --- | --- |
|  | **O-MicroD** | | | **MicroD** | | | **RT** | | |
|  | Species | Concentration | CV | Species | Concentration | CV | Species | Concentration | CV |
| Alkali metal | 3 | 164.82 ± 191.96 | 6.08 ± 2.36 | 4 | 269.34 ± 325.55 | 63.02 ± 37.08 | 2 | 265.58 ± 24.85 | 105.35 ± 61.38 |
| Alkaline earth metal | 4 | 22.1 ± 29.82 | 6.48 ± 5.53 | 3 | 24.17 ± 30.58 | 74.62 ± 18.26 | 4 | 19.5 ± 23.63 | 38.63 ± 17.33 |
| Transition metal | 16 | 13.62 ± 32.09 | 23.19 ± 35.99 | 16 | 19.04 ± 41.54 | 59.77 ± 21.94 | 10 | 15.16 ± 35.39 | 21.32 ± 15.43 |
| Post-transition metal | 4 | 1.62 ± 2.97 | 17.61 ± 5.00 | 4 | 84.96 ± 169.31 | 65.84 ± 29.07 | 1 | 0.35 | 41.37 |
| Metalloid | 2 | 0.57 ± 0.67 | 11.77 ± 7.48 | 1 | 0.44 | 36.33 | NA | NA | NA |
| Non-metal | 2 | 63.49 ± 89.45 | 14.86 ± 1.17 | 2 | 71.18 ± 100.2 | 14.75 ± 5.99 | 1 | 0.18 | 34.99 |
| Actinide | 1 | 0.05 | 3.26 | 2 | 0.09 ± 0.02 | 63.45 ± 42.9 | 1 | 0.06 | 25.33 |
| Lanthanide | 6 | 0.003 ± 0.003 | 58.35 ± 33.96 | 12 | 0.09 ± 0.15 | 78.65 ± 16.78 | 6 | 0.002 ± 0.002 | 51.8 ± 22.25 |
| Total identified metals | 38 | 24.62 ± 68.04 | 23.49 ± 31.09 | 44 | 44.05 ± 126.96 | 64.37 ± 25.68 | 25 | 30.45 ± 75.07 | 39.63 ± 30.55 |

| **T90** | | | **UltraS** | | |  |
| --- | --- | --- | --- | --- | --- | --- |
| Species | Concentration | CV | Species | Concentration | CV |  |
| 3 | 98.55 ± 88.14 | 22.48 ± 21.99 | 1 | 90.11 | 38.9 |  |
| 4 | 14.32 ± 18.64 | 9.41 ± 7.94 | 2 | 8.62 ± 11.48 | 10.38 ± 2.47 |  |
| 16 | 9.1 ± 22.76 | 24.32 ± 21.16 | 11 | 11.68 ± 25.67 | 21.67 ± 21.51 |  |
| 4 | 1.63 ± 3.04 | 32.23 ± 20.81 | 1 | 0.31 | 29.34 |  |
| 2 | 0.47 ± 0.56 | 34.3 ± 38.11 | NA | NA | NA |  |
| 2 | 65.57 ± 92.5 | 18.17 ± 8.77 | 1 | 0.19 | 25.99 |  |
| 1 | 0.05 | 14.11 | 1 | 0.06 | 9.48 |  |
| 6 | 0.002 ± 0.002 | 36.87 ± 9.65 | 5 | 0.0007±0.0004 | 70.9 ± 33.55 |  |
| 38 | 16.77 ± 40.98 | 25.35 ± 19.17 | 22 | 10.74 ± 25.78 | 32.61 ± 30.40 |  |
| Data are presented as mean ± SD; O-MicroD: Ordinary microwave oven digestion; MicroD: Microwave digestion; RT: Room temperature; T90: Temperature 90℃; UltraS: Ultrasonic water bath; CV: coefficient of variance. | | | | | | |

| **Table S4. The quantitative concentrations (μg/L) of identified metals in human hairs across the five different digestion methods.** | | | | | | |
| --- | --- | --- | --- | --- | --- | --- |
| Metals | Classes | O-MicroD | MicroD | RT | T90 | UltraS |
| Cs | Alkali metal | < LOD | 0.0232 ± 0.0088 | < LOD | < LOD | < LOD |
| K | Alkali metal | 375.7433 ± 23.8464 | 419.6287 ± 242.3548 | 248.0069 ± 153.6286 | 170.3261 ± 8.8371 | < LOD |
| Na | Alkali metal | 118.3665 ± 9.8290 | 214.7949 ± 69.4054 | 72.9563 ± 31.5937 | 125.1595 ± 59.1109 | 90.1138 ± 35.0580 |
| Rb | Alkali metal | 0.3463 ± 0.0125 | 0.4051 ± 0.1597 | < LOD | 0.1789 ± 0.0269 | < LOD |
| Ba | Alkaline earth metal | 164.8187 ± 0.0995 | 3.7382 ± 2.4574 | 0.5892 ± 0.1466 | 0.4903 ± 0.0934 | 0.5073 ± 0.0615 |
| Ca | Alkaline earth metal | 191.9611 ± 2.4532 | < LOD | 50.3054 ± 15.4934 | 40.278 ± 0.7775 | < LOD |
| Mg | Alkaline earth metal | 0.6787 ± 1.0838 | 59.3228 ± 37.0699 | 25.7581 ± 16.4532 | 15.4598 ± 1.9608 | 16.7359 ± 1.4449 |
| Sr | Alkaline earth metal | 64.2328 ± 0.0329 | 9.4478 ± 9.0343 | 1.3355 ± 0.4667 | 1.0402 ± 0.0413 | < LOD |
| Ag | Transition metal | 22.2309 ± 0.0016 | 0.0972 ± 0.0585 | < LOD | 0.0822 ± 0.0220 | 0.1951 ± 0.1251 |
| Cd | Transition metal | < LOD | 0.0374 ± 0.0181 | < LOD | < LOD | < LOD |
| Co | Transition metal | 1.2706 ± 0.0054 | 0.1572 ± 0.0689 | 0.0477 ± 0.0045 | 0.0791 ± 0.0064 | 0.0516 ± 0.0021 |
| Cr | Transition metal | 22.1033 ± 0.5297 | 11.3679 ± 4.3094 | 3.036 ± 0.1985 | 7.1071 ± 1.9986 | 4.2320 ± 0.2939 |
| Cu | Transition metal | 29.8213 ± 2.4667 | < LOD | 8.1952 ± 0.3740 | 8.3219 ± 0.0565 | 8.3009 ± 0.3806 |
| Fe | Transition metal | 0.0542 ± 3.9444 | 120.9288 ± 56.7181 | 25.2546 ± 1.9318 | 41.0435 ± 11.7181 | 29.2897 ± 2.316 |
| Hg | Transition metal | 0.1517 ± 0.0431 | 0.0992 ± 0.0400 | 0.1654 ± 0.0339 | 0.1884 ± 0.0136 | < LOD |
| Mn | Transition metal | 13.4465 ± 0.0537 | 2.4683 ± 1.5141 | < LOD | 0.4456 ± 0.0699 | < LOD |
| Mo | Transition metal | 11.9472 ± 0.0210 | 0.2467 ± 0.1094 | 0.0822 ± 0.0150 | 0.0866 ± 0.0137 | 0.0722 ± 0.0086 |
| Ni | Transition metal | 70.0498 ± 0.2952 | < LOD | 1.4182 ± 0.3562 | 2.7680 ± 0.4174 | 1.7221 ± 0.3509 |
| Re | Transition metal | < LOD | 0.0672 ± 0.0213 | < LOD | 0.0069 ± 0.0061 | 0.0101 ± 0.0052 |
| Ta | Transition metal | 0.3986 ± 0.0014 | 0.0077 ± 0.0034 | < LOD | 0.0264 ± 0.0070 | 0.0237 ± 0.0105 |
| Ti | Transition metal | 0.7685 ± 0.2649 | 45.6805 ± 46.5299 | < LOD | < LOD | < LOD |
| V | Transition metal | 0.1344 ± 0.0070 | 0.5468 ± 0.4367 | 0.0335 ± 0.0160 | 0.0448 ± 0.0089 | 0.0242 ± 0.0045 |
| W | Transition metal | 5.5175 ± 0.1576 | < LOD |  | 0.0139 ± 0.0066 | < LOD |
| Y | Transition metal | 0.0065 ± 0.0010 | 0.1179 ± 0.1152 | 0.0009 ± 0.0004 | < LOD | < LOD |
| Zn | Transition metal | 0.5511 ± 5.8743 | 121.7873 ± 94.1980 | 113.3784 ± 32.7876 | 85.3275 ± 1.5147 | 84.5193 ± 3.4377 |
| Zr | Transition metal | 0.0715 ± 0.0055 | 0.9972 ± 0.8412 | < LOD | 0.0237 ± 0.0053 | < LOD |
| Nb | Transition metal |  | 0.0661 ± 0.0371 | < LOD | 0.0060 ± 0.0022 | < LOD |
| Al | Basic-metal | 0.1056 ± 0.8111 | 338.9205 ± 336.3078 | < LOD | 6.1769 ± 3.3592 | < LOD |
| Ga | Basic-metal | 0.0029 ± 0.0020 | 0.1634 ± 0.1283 | < LOD | 0.0055 ± 0.0025 | < LOD |
| Pb | Basic-metal | 114.6981 ± 0.0518 | 0.7404 ± 0.3906 | 0.3524 ± 0.1458 | 0.3286 ± 0.0545 | 0.3105 ± 0.0911 |
| Tl | Basic-metal | 0.0200 ± 0.0007 | 0.0271 ± 0.0089 | < LOD | 0.0024 ± 0.0003 | < LOD |
| As | Metalloid | 13.6203 ± 0.0063 | 0.4365 ± 0.1586 | < LOD | 0.0707 ± 0.0052 | < LOD |
| Ge | Metalloid | 32.0931 ± 0.1786 | < LOD | < LOD | 0.8624 ± 0.5282 | < LOD |
| P | Non-metal | 6.0632 ± 19.8800 | 142.0331 ± 14.9431 | < LOD | 130.9723 ± 15.6736 | < LOD |
| Se | Non-metal | 0.0091 ± 0.0337 | 0.3281 ± 0.0623 | 0.1806 ± 0.0632 | 0.1592 ± 0.0388 | 0.1947 ± 0.0506 |
| Ce | Lanthanide | 0.3922 ± 0.0035 | 0.5098 ± 0.3559 | < LOD | 0.0061 ± 0.0012 | < LOD |
| Er | Lanthanide | 0.0032 ± 0.0007 | 0.0144 ± 0.0126 | 0.0011 ± 0.0003 | < LOD | < LOD |
| La | Lanthanide | 1.6169 ± 0.0012 | 0.2124 ± 0.2030 | < LOD | 0.0025 ± 0.0008 | < LOD |
| Nd | Lanthanide | 2.9698 ± 0.0019 | 0.1592 ± 0.1525 | 0.005 ± 0.0023 | 0.0034 ± 0.0015 | 0.0015 ± 0.0007 |
| Pr | Lanthanide | 0.0972 ± 0.0006 | 0.0438 ± 0.0396 | < LOD | 0.0011 ± 0.0005 |  |
| Tm | Lanthanide | 1.0471 ± 0.0001 | 0.0044 ± 0.0020 | 0.0004 ± 0.0002 | < LOD | 0.0005 ± 0.0004 |
| Dy | Lanthanide | < LOD | 0.0268 ± 0.0220 | < LOD | 0.0005 ± 0.0002 | < LOD |
| Eu | Lanthanide | < LOD | 0.0079 ± 0.0042 | < LOD | < LOD | < LOD |
| Gd | Lanthanide | < LOD | 0.0269 ± 0.0243 | 0.0012 ± 0.0008 | < LOD | 0.0007 ± 0.0003 |
| Ho | Lanthanide | < LOD | 0.0063 ± 0.0041 | 0.0006 ± 0.0002 | < LOD | 0.0005 ± 0.0003 |
| Sm | Lanthanide | < LOD | 0.0259 ± 0.0234 | < LOD | < LOD | 0.0004 ± 0.0005 |
| Yb | Lanthanide | < LOD | 0.0161 ± 0.0126 | 0.0008 ± 0.0007 | 0.0005 ± 0.0002 | < LOD |
| Th | Actinide | < LOD | 0.0724 ± 0.0679 | 0.0604 ± 0.0153 | < LOD | < LOD |
| U | Actinide | 0.5722 ± 0.0017 | 0.1039 ± 0.0344 | < LOD | 0.0482 ± 0.0068 | 0.0559 ± 0.0053 |
| Data are presented as mean ± SD; | | | | | | |
| O-MicroD: Ordinary microwave oven digestion; MicorD: Microwave digestion; RT: Room temperature; T90: Temperature 90℃; UltraS: Ultrasonic | | | | | | |
| water bath; LOD: limit of detection. | | | | | | |


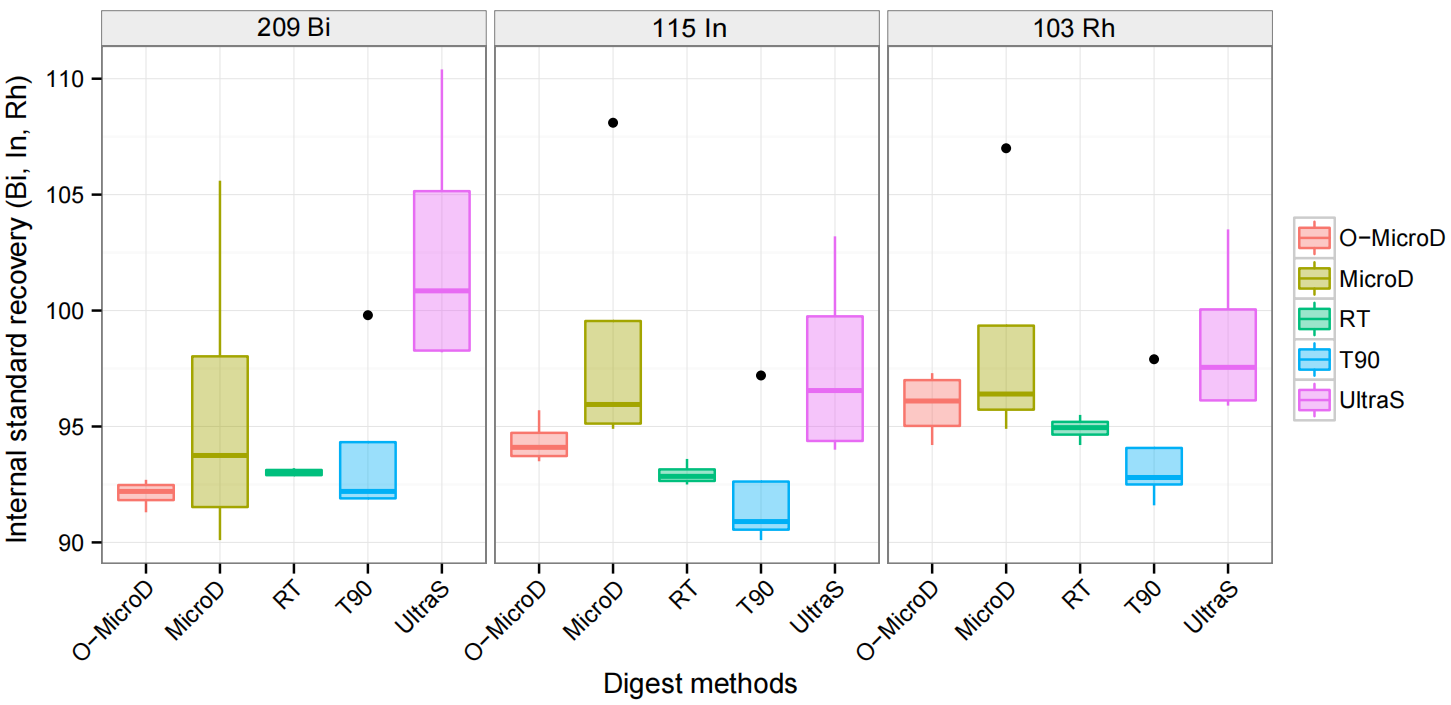


**Figure S1.** The extraction efficiency of internal standard elements (Bi, In, Rh) in five different digestion methods for human hair. Four experimental replicates were measured for each digestion method. The distributions of the boxplots are minimum, 25th percentile, median, 75th percentile, and maximum (from bottom to top). Dots are outliers (above or below the median by more than 1.5 times the interquartile range).


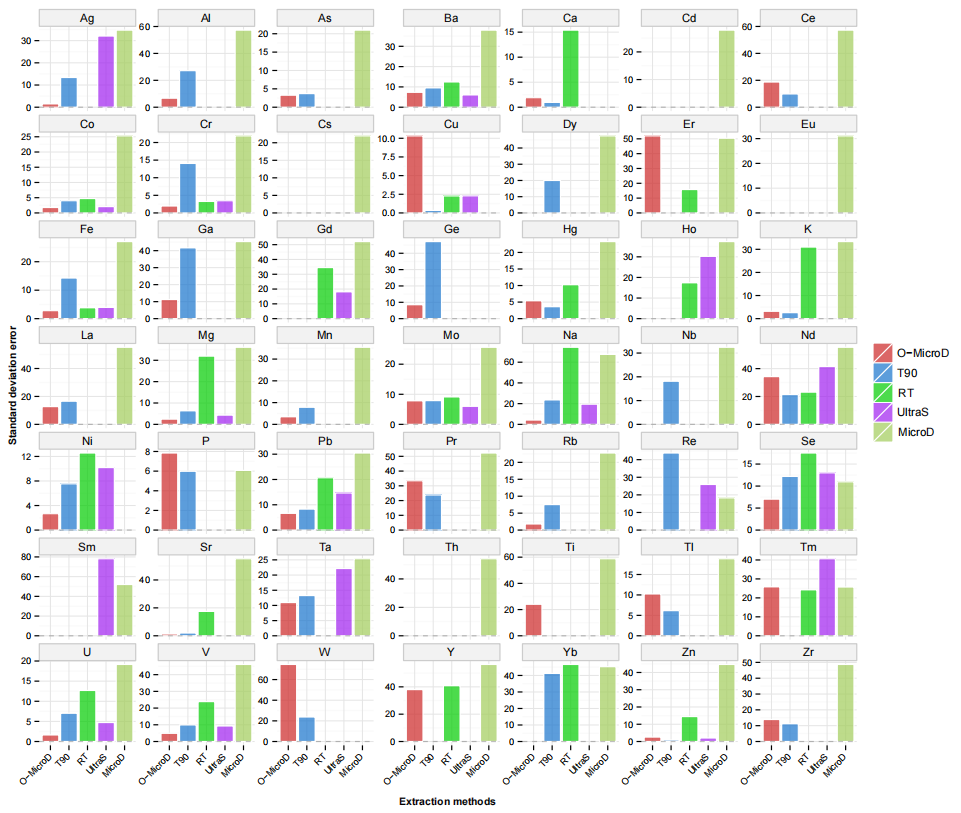


**Figure S2.** The bar graphs show the coefficient of variation (%) of each metal across five different digestion methods for human hair.
